# Deciphering Nucleic Acid Binding Proteome of Mouse Immune Organs Reveals Hub Proteins for Aging

**DOI:** 10.1016/j.mcpro.2023.100611

**Published:** 2023-06-28

**Authors:** Huiyu Wang, Yan Zhang, Zeyuan Wang, Lu Zhang, Miao Guo, Chengxi Cao, Hua Xiao

**Affiliations:** 1 State Key Laboratory of Microbial Metabolism, Joint International Research Laboratory of Metabolic & Developmental Sciences, School of Life Sciences and Biotechnology, Shanghai Jiao Tong University, Shanghai, China; 2 School of Pharmacy, Shanghai Jiao Tong University, Shanghai, China; 3 Department of Instrument Science and Engineering, School of Electronic Information and Electrical Engineering, Shanghai Jiao Tong University, Shanghai, China

**Keywords:** NABPs, proteomics, aging, WGCNA, immune system

## Abstract

Profiling the nucleic acid–binding proteins (NABPs) during aging process is critical to elucidate its roles in biological systems as well as transcriptional and translational regulation. Here, we developed a comprehensive strategy to survey the NABPs of mouse immune organs by using single cell preparation and selective capture technology-based proteomics. Our approach provided a global view of tissue NABPs from different organs under normal physiological conditions with extraction specificity of 70 to 90%. Through quantitative proteomics analysis of mouse spleen and thymus at 1, 4, 12, 24, 48, and 72 weeks, we investigated the molecular features of aging-related NABPs. A total of 2674 proteins were quantified in all six stages, demonstrating distinct and time-specific expression pattern of NABPs. Thymus and spleen exhibited unique aging signatures, and differential proteins and pathways were enriched across the mouse lifespan. Three core modules and 16 hub proteins associated with aging were revealed through weighted gene correlation network analysis. Significant candidates were screened for immunoassay verification, and six hub proteins were confirmed. The integrated strategy pertains the capability to decipher the dynamic functions of NABPs in aging physiology and benefit further mechanism research.

## Highlights

• A comprehensive strategy was developed to decipher the NABPome of solid organs.
• Single cell preparation and Ti^4+^-IMAC capture technology were used for proteomics.
• Unique molecular features of aging were revealed from mouse spleen and thymus.
• Coexpression networks were constructed to explore age-associated modules/proteins.
• Sixteen age-associated hub NABPs were revealed and 6 were verified by immunoassay.

## Graphical Abstract

**Figure:**
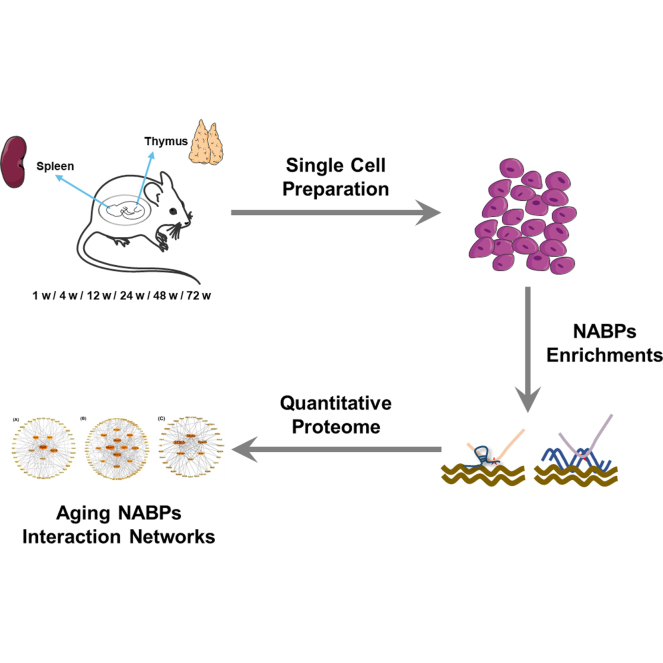

## In Brief

We developed a robust strategy for in-depth analysis of organ NABPs, which greatly overcame the contamination of blood cells and plasma proteins and increased the number and intensity of identified tissue NABPs. Molecular features of aging-related NABPs from mouse spleen and thymus were identified as hub proteins by quantitative proteomics and WGCNA analysis. Our data collectively support the notion that aging is associated with an altered ability of NABPs to bind to nucleic acid, highlighting the efficacy and broad application of our strategy.

Aging is one of the critical risk factors for most chronic diseases characterized by a decline in physiological function of all organs (1). Life expectancy is generally influenced by many gene–protein interactions. Gene expression patterns, which include nucleic acids, binding proteins, in turn, are also dramatically affected by aging (2, 3). Age-related gene expression patterns are potently regulated at the replication and transcription levels through the action of chromatin remodeling factors, such as MRG (4) (mortality factor on chromosome 4-related gene), HDACs (5) (histone deacetylases), and transcription factors such as FOXO (6) (fork head box), and peroxisome proliferator-activated receptor (PPAR) (7). These proteins, known as nucleic acid–binding proteins (NABPs), are expressed at different times to enable cells to perform essential functions during development and to respond to a series of stresses (8).

Despite the considerable advances in understanding the important role of NABPs in aging, the dynamic changes of NABPs during aging process remain unclear, which calls for powerful approach to decipher both the DNA-binding proteome and the RNA-binding proteomes. Organic phase-based separation methods have been well established to interrogate the RNA-binding proteome, and affinity purification methods have been successfully employed to enrich the chromatin-binding proteome (9). We have recently developed a selective and powerful tool named Titanium ion–immobilized metal-affinity chromatography (Ti^4+^-IMAC) capture method for the comprehensive and dynamic characterization of nucleic acid–binding proteome (NABPome) at living cell level (10). Through utilizing formaldehyde crosslinking, NABPs were covalently linked to nucleic acids. The nucleic acids–protein complexes were further denatured by organic solvent and captured by Ti^4+^-IMAC. However, the extraction of NABPs from solid tissues still faces many technical challenges, such as high abundant plasma protein interference, low extraction specificity, and difficult crosslinking operations. Therefore, it is necessary to develop an efficient and specific method for the *in situ* extraction and large-scale identification of tissue NABPs.

During aging, the immune system gradually loses its ability to respond effectively to infections, autoimmune diseases, and cancer. In particular, the thymus is an essential immune organ that is responsible for generating T cells and involved in adaptive immunity (11), while the spleen is a key immune organ that continuously shows an active role in maintaining immune functions, red blood cell turnover, and microbial defense (12). However, the thymus will gradually lose its architecture and function during aging, leading to lymphocyte depletion and deterioration of thymic stromal microenvironment. The spleen which has an innate capacity to regenerate does not show many phenotypic changes during aging (13). Comparative NABPome analysis of the two immune organs during aging has the potential to help us better understand the complex mechanisms of aging.

Mice (*Mus musculus*) are routinely used as laboratory animals in biomedical research and mammalian model for studying human health and disease (14). The lifespan of mice is relatively short, with one adult mouse month equivalent to approximately three human years (15). In mice, young mouse is defined as 1- to 6-weeks-old, and senescence mouse is started from 48 to 56 weeks. The intermediate stage corresponds to adulthood between 6 and 8 weeks to 48 weeks. This allows the longest lifespan studies to be conducted within the timeline of a typical research project (16).

In this study, we developed a specific tissue NABPs enrichment strategy to achieve an in-depth analysis of organ NABPome. The new strategy consisted of four procedures, including single cell preparation, formaldehyde crosslinking, organic denaturation, and Ti^4+^-IMAC capture, which greatly increased the number and intensity of identified NABPs in different mouse organs. With the help of quantitative proteomics, we applied this strategy to study the NABPs expression changes in thymus and spleen during aging. Among identified NABPs, we analyzed the NABPs network of mouse immune organs during different aging stages, allowing in-depth data mining and exploring the roles of NABPs in aging processes. Our integrated analysis strategy revealed dysregulated hubs NABPs and critical pathways for aging research.

## Experimental Procedures

### Experimental Design and Statistical Rationale

To evaluate the NABPs enrichment efficiency of the Ti^4+^-IMAC method, total proteome and NABPome from different organs with three biological replicates were analyzed and compared through label-free quantification. The significantly enriched proteins were selected using two-sided Student’s *t* test with fold change >2 and *p* value <0.05. To elucidate the mechanism by which NABPs regulate the aging process, the proteomes of NABPs derived from thymus and spleen at the age of 1, 4, 12, 24, 48, and 72 weeks (n = 4) were analyzed by label-free quantification–based proteomics. The comparisons of quantitative data between multiple groups were assessed by using two-sided Student’s *t* test, and *p* < 0.05 was considered as statistically significant.

### Animals and Mice Organ Collection

All mice were housed (four to five animals per cage) with a 12/12 h light/dark cycle, with food and water ad libitum. All animal experiments were performed in accordance with the National Institutes of Health Guide for the Care and Use of Laboratory Animals, with procedures approved by the Biological Research Ethics Committee of Shanghai Jiao Tong University (approval number 20211025–01). Mice were sacrificed by cervical dislocation.

### Single Cell Preparation

For single cell preparation, kidney, spleen, and thymus cells were isolated by using mechanical digestion method. Briefly, the organ was transferred to a sterile culture dish containing 5 ml medium (Dulbecco's modified Eagle's medium containing 10% fetal bovine serum) for subsequent isolation. The flat end of the plunger from a sterile syringe was taken out and crush the organs several times in gentle circular motions. This action busted the organ and released the single cell. Since the lung cells were difficult to separate from lung tissue, a combination of mechanical digestion method and enzymatic digestion method was used to prepare single cells. Briefly, mouse lung tissues were minced into small pieces and then digested in 3 mg/ml collagenase I with 200 μg/ml DNase I for 45 min. Dissociated cells in suspension were passed through a 70 μm strainer and centrifuged at 300*g* for 5 min at 4 °C. Red blood cell lysis reagent (Thermo Fisher) was used to deplete erythrocytes. All procedures were performed on ice. Cell viability was assessed by propidium iodide staining and flow cytometry detection (17). Briefly, cells were washed twice and then incubated with propidium iodide staining solution for 20 min at room temperature in darkness according to the manufacturer’s protocol (Sangon Biotech). Flow cytometry analysis was performed by FACSCanto II flow cytometer (BD Biosciences), and acquired data were analyzed using the FlowJo software.

### Isolation of NABP From Mouse Organ at Different Age Stage

NABPs were isolated using our recently developed Ti^4+^-IMAC capture approach (10). Briefly, the prepared single cells were washed twice with PBS and crosslinked by 1% formaldehyde (10 ml) for 10 min at room temperature. After crosslinking, 5 × 10^6^ cells were lysed by 1 ml TRIZOL reagent and incubated at room temperature for 5 min, and 200 μl of chloroform was then added and vortexed at maximum speed for 15 s. The interphase and organic phase containing NAs-protein complexes were precipitated by isopropanol at room temperature for 10 min. The precipitated crude NAs–protein complexes were further resuspended in 100 μl 0.1% SDS with proteinase cocktail and RNase inhibitor, then incubated at 55 °C for 20 min and sonicated for 2 min (250 w, working for 2 s and pausing for 10 s). Crude NAs–protein complexes were further enriched by mixing with Ti^4+^-IMAC material for 1 h. To remove nonspecific-binding proteins, the pellet was washed twice with 500 μl wash buffer (200 mM NaCl, 0.1% TFA, 0.1% NP-40, 10 mM EDTA) for 20 min. The enriched NAs–protein complexes were eluted from Ti^4+^-IMAC with 10% ammonium hydroxide (NH_3_·H_2_O) by vortexing and ultrasonication, then decrosslinked by incubation at 65 °C for 6 h.

### NABPs Digestion and LC-MS/MS Analysis

50 μg NABPs were reduced with 20 mM DTT for 60 min and alkylated with 100 mM iodoacetamide for 40 min in dark. Filter-aided sample preparation approach (18) was used for SDS removal, and NABPs was further digested with 1 μg trypsin at 37 °C for 16 h. The obtained peptides were desalted by using Ziptip C18 (Millipore). The NABPs samples were analyzed on a Q Exactive Plus MS (Thermo Fisher Scientific) interfaced with an Easy-nLC 1000 nanoflow LC system (Thermo Fisher Scientific). Peptides were first loaded and separated by a C18 analytical column (Jupiter 3 μm C18, 50 μm × 25 cm, Phenomenex). The mobile phase consisted of solvent A (0.1% formic acid in water) and solvent B (0.1% formic acid in 95% acetonitrile). A flow rate of 300 nl/min and 120 min gradient was applied as follows: 5% B (0–5 min), 5 to 22% B (6–95 min), 22 to 32% B (96–110 min), 32 to 90% B (111–113 min), and 90% B (114–120 min). The mass spectrometer’s scan ranged from 350 to 2000 m/z with the MS resolution of 70,000. The resolution of MS/MS was 17,500.

### Proteomic Data Processing

Quantitative data analysis was performed by MaxQuant (v1.6.1.2) using raw data files of MS/MS spectra searched against the UniProtKB mouse database (release 2021_12_26, 27,414 entries) (19). An initial search was set at a precursor mass window of 6 ppm. The search followed by an enzymatic cleavage rule of Trypsin/P. Maximal two missed cleavage sites were allowed with a mass tolerance of 20 ppm for fragment ions. Carbamidomethylation of cysteines was defined as fixed modification, while protein N-terminal acetylation and methionine oxidation were defined as variable modifications for database searching. “Unique+razor” peptides were used for quantification. The cutoff of false discovery rate by using a target-decoy strategy was 1% for both proteins and peptides. R/Bioconductor package DEP (v.1.17.1) and Limma (v.3.24.15) provides an integrated analysis workflow for differential protein expression. We eliminated the proteins that were identified by site, considered as potential contaminants, or had reversed sequences. Only proteins with at least two unique peptides were considered as identified while only proteins with LFQ intensity in at least two of the four replicates were defined as quantified. Variance stabilizing normalization (vsn) was performed to ensure that each sample had the same distribution. Missing values were handled by random values drawn from normal distribution of 1.8 SD apart with a width of 0.3.

### Functional Enrichment Analysis of Differentially Expressed Proteins

To gain further insight into the biological implications of NABPome, we performed gene set variation analysis (GSVA) analysis to identify the pathway alterations that among different mouse organs and age stages, using the R/Bioconductor package GSVA (20) (v.1.16.0). GSVA analysis requires the following two main input arguments: the gene expression data and a collection of gene sets. In this study, the gene sets were obtained from the MSigDB database v.5.2 (http://software.broadinstitute.org/gsea/msigdb/index.jsp). Another input was the expression matrix of the signature proteins. The gene identifiers were unified by the UniProt ID and mapped to the Human Genome Nomenclature Committee’s HUGO symbol (http://www.genenames.org/). The protein expression matrix was subjected to the GSVA algorithm to calculate the ssGSEA scores for each gene set with at least 10 overlapping genes.

### Weighted Gene Correlation Network Analysis

The co-expression network analysis was specifically performed for aging samples using R package weighted gene correlation network analysis (WGCNA) which modified slightly for protein co-abundance analysis (21). We used log-transformed LFQ protein intensity to construct the expression matrix. First, the sample was clustered to assess the presence of any obvious outliers. Then, the optimal soft threshold for adjacency computation was determined, and hierarchical clustering and dynamic tree cut function were used to detect modules. Next, gene significance and module membership were calculated to relate modules to clinical traits. Finally, the corresponding module gene information was extracted and the network of eigengenes was visualized. The proteins in important eigengenes with a gene significance > 0.32 and a module membership > 0.85 were used to construct module networks. The interactive relationships between the proteins in each module were further visualized using the Cytoscape software (MCODE, Cytoscape, and CytoHubba), and proteins with the highest connectivity (connectivity degree>20) were used as hub proteins in each network.

### Western Blot Analysis

Enriched NABPs was quantified by using BSA method. 20 μg protein lysate was loaded onto SDS-PAGE gels and then transferred to polyvinylidene fluoride membranes (Pall Life Sciences). Polyvinylidene fluoride membranes were blocked with 5% no-fat milk in room temperature and incubated overnight at 4 °C with the following primary antibodies: anti-RPL6 antibody (1:1000) (A15094, Abclona), anti-RPL18 antibody (1:1000) (A10720, Abclona), anti-HDAC2 antibody (1:1000) (A19626, Abclona), anti-MCM2 antibody (1:1000) (A20699, Abclona), anti-MCM5 antibody (1:1000) (A5008, Abclona), anti-MCM7 antibody (1:1000) (A1138, Abclona), anti-UPF1 antibody (1:1000) (A5071, Abclona), anti-SBDS antibody (1:1000) (A0796, Abclona), anti-RPS25 antibody (1:1000) (A5008, Abclona), and anti-MCTS1 antibody (1:1000) (A9061, Abclona). The membranes were further washed and incubated with HRP-conjugated anti-rabbit IgG secondary antibody for 1 h at room temperature. Finally, the membranes were briefly washed and visualized with a Tanon 5200 multi-imaging system (Tanon).

## Results

### Ti^4+^-IMAC Capture Facilitated NABPs Identification in Mouse Organs

**Figure 1:**
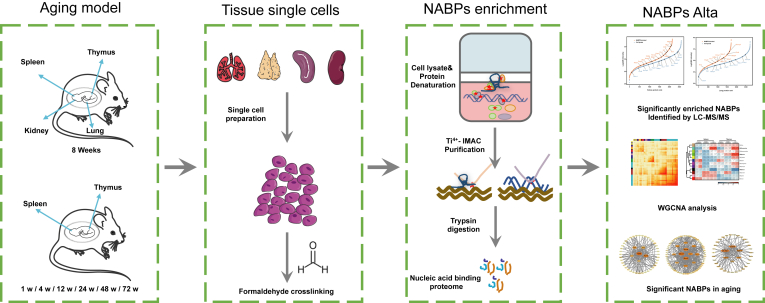
**The workflow for the analysis of NABPome in different mouse organs at different age**. Different mouse organs are manually isolated and subjected to single cell preparation. After formaldehyde crosslinking, cells were lysate by TRIZOL reagent and further captured by Ti^4+^-IMAC. Nucleic acid binding proteome were deciphered by mass spectrometry technology and WGCNA analysis. NABPome, nucleic acid–binding proteome; NABP, nucleic acid–binding protein; Ti^4+^-IMAC, titanium ion–immobilized metal-affinity chromatography; WGCNA, weighted gene correlation network analysis.

**Figure 2:**
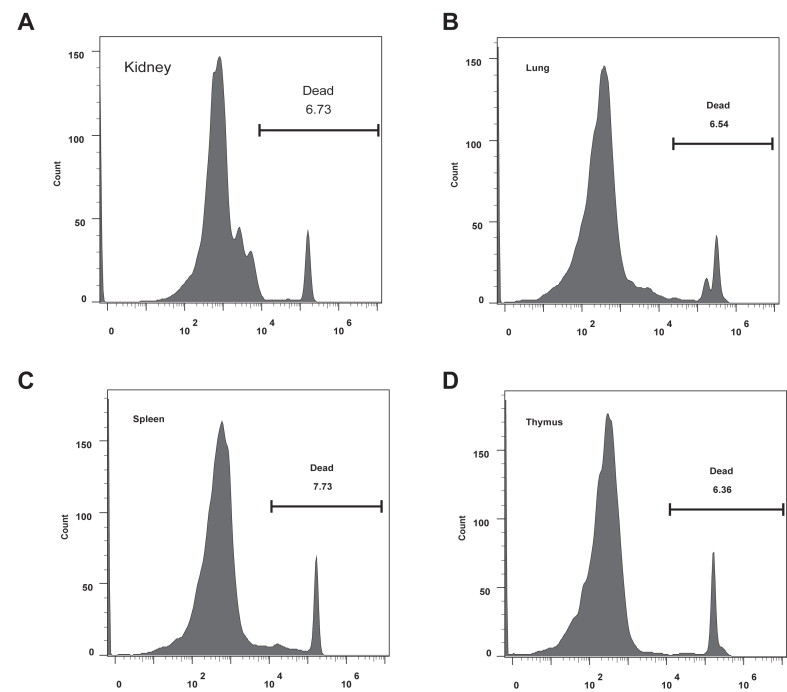
**Flow cytometry assays determined tissue cell viability after single cell preparation.***A*, kidney; *B*, lung; *C*, spleen; *D*, thymus.

It is challenging to provide a comprehensive overview of organ NABPome in a simple and robust manner. Here, we have developed integrated strategy to capture the whole organ NABPs under physiological aging conditions (Fig. 1). In order to overcome the problems of low formaldehyde crosslinking efficiency and high abundant plasma protein interference in conventional tissue lysate protocol, we prepared single cells from fresh kidney, lung, spleen, and thymus tissues with the help of mechanical digestion and enzyme digestion method. Our experimental results showed that single living cells were successfully extracted from these organ tissues (Fig. 2). Through utilizing formaldehyde crosslinking in living cells, NABPs were covalently linked to nucleic acids *in situ*. Isolated cells were further lysed by TRIZOL regents which could denature nucleic acid–protein complex. To avoid RNA degradation due to overcrosslinking, our previous study optimized that 0.2% FA was sufficient for NAs-protein crosslinking and highly concentrated RNA could be recovered from the crosslinked complex (10).

**Figure 3:**
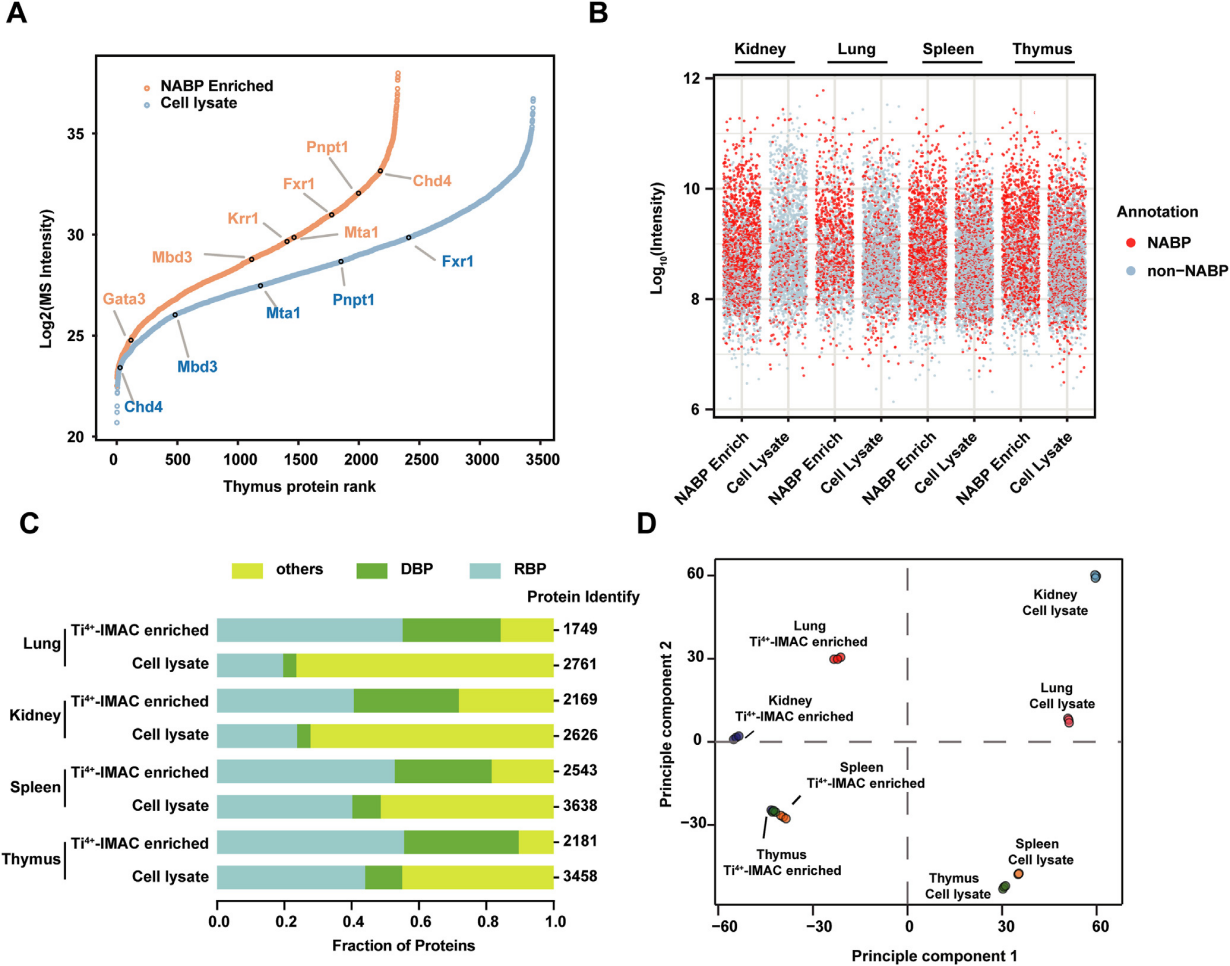
**Quantitative evaluation of NABPs enrichment in different mouse organs**. *A,* dynamic range plot of proteins identified in Ti^4+^-IMAC capture group and total tissue lysate group. Nucleosome remodeling and deacetylase and K-homology type protein family are listed and sorted by their abundance (Log_2_ MS intensity). *B*, distributions of the relative abundances of NABPs and non-NABPs in different mouse organs of Ti^4+^-IMAC–capture group and total tissue lysate group. *C*, DBPs and RBPs fraction in Ti^4+^-IMAC–capture group and total tissue lysate group of different mouse organs. The proportion of DBPs, RBPs, and other proteins were determined based on intensity of enriched NABPs and cell lysate. *D,* PCA of the proteomics datasets. Each data point represents a single replicate. DBP, nucleosome remodeling and deacetylase; NABP, nucleic acid–binding protein; RBP, K homology domain; Ti^4+^-IMAC, titanium ion–immobilized metal-affinity chromatography.

We performed quantitative proteomics analysis for the Ti^4+^-IMAC captured NABPome and the total tissue lysate proteome to assess the efficiency of NABPs enrichment (supplemental Table S1). The dynamic range plots exhibit the expression of classical NABPs in total tissue lysate group and Ti^4+^-IMAC capture group (Figs. 3*A* and S1). The detected nucleosome remodeling and deacetylase (DBPs) and K homology domain (RBPs) are listed in supplemental Table S1. NABPs such as Chd4, Mbd3, Krr1, Gata3, and Mta1 were undetected or with low MS intensity in the total tissue lysate group, whereas they were significantly enriched in the Ti^4+^-IMAC capture group. For different mouse organs, the protein intensity fraction of Gene Ontology (GO) annotated NABPs reached 70 to 90% in the Ti^4+^-IMAC capture group, compared to 30 to 50% in the total tissue lysate group (Fig. 3*B*). In addition, an overall increase was observed in the proportion of proteins classified as RBP or DBP in Ti^4+^-IMAC capture group, which reached 20% and 30%, respectively, compared with the tissue lysate group (Fig. 3*C* and supplemental Table S2). The specific characteristics and cell-type composition of the mouse organs are likely to influence the efficiency of the NABPs enrichment protocol. Enrichment volcano plot and Pearson correlation coefficients (supplemental Fig. S2) demonstrated the reproducibility of the biological replicates and confirmed the robustness of our biochemical and proteomic methodology. Principal component analysis (Fig. 3*D*) demonstrated a clear distinction between the proteomes of NABPs and cell lysate, which further highlighting the necessity of NABPs enrichment. Therefore, the single cell separation and Ti^4+^-IMAC capture strategy facilitated the specific enrichment of NABPs, which could be utilized for the analysis of NABPome in diverse mouse organs.

### NABPome Reveals Unique Biological Characteristics in Distinct Mice Organs

We performed GO analysis to assess the characteristics of proteins obtained from the enriched group and cell lysate group from different mouse organs. GO analysis showed a distinct enrichment of proteins associated with ‘DNA binding’, ‘RNA splicing’, ‘mRNA-containing ribonucleoprotein complex’, and ‘DNA metabolic process’ in the Ti^4+^-IMAC capture group (supplemental Fig. S3*A*). The total tissue lysate group mainly contained proteins involved in ‘protein folding’, ‘cell–cell adhesion’, and ‘cadherin binding (supplemental Fig. S3*B*). These data reconfirmed that NABPs was highly enriched by Ti^4+^-IMAC capture. Notably, protein changes in the Ti^4+^-IMAC–enriched group did not correlate with their total protein abundance in many cases, suggesting that Ti^4+^-IMAC method was attributed to a change in DNA/RNA-binding rather than the overall protein expression (supplemental Fig. S3*C* and supplemental Table S3). To further understand the biological significance of these identified NABPs, we performed GSVA analysis to reveal NABPs-associated core pathways among four different mouse organs.

Of the 1921 NABPs identified from kidney, lung, spleen, and thymus, 607 proteins were differentially expressed, corresponding to 25 core pathways. Through knowledge-based annotation, we refined these pathways to the following five biological function categories: cell development, signal transduction, immune response, cell proliferation, and metabolism. The dysregulation of NABPs expression in different tissues led to the activation of different biological functions. For instance, significant changes were observed in the thymus and spleen that linked to ‘immune response’; changes of ‘oxidative stress’ and ‘lipid metabolism’ were observed in the lung, and ‘cell cycle mitotic’ changes were defined across the kidney (supplemental Fig. S3*D*). The differentially expressed NABPs and pathways in different mouse organs suggest that NABPs play different nucleic acid binding capabilities in different organs and may be the driving forces in tissue development and tissue identity maintenance.

### Quantitative NABPome Characterization of Aging Mouse Organs

**Figure 4:**
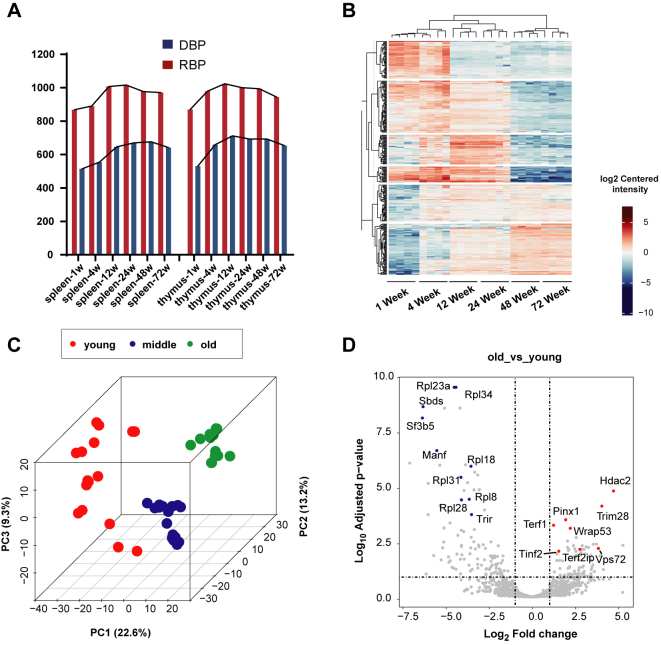
**Effect of age on protein expression levels**. *A*, the number of DBPs and RBPs in mouse spleen and thymus in different age stages. *B*, the heatmap of the 1698 significantly age-associated NABPs reveals changing expression profiles across aging. *C*, PLS analysis of age-associated proteins were classified into three age groups: young stage (1 week and 4 weeks), middle-aged stage (12 weeks and 24 weeks), and old mature stage (48 weeks and 72 weeks). *D*, volcano plots of proteins in young and old stage. DBP, nucleosome remodeling and deacetylase; NABP, nucleic acid–binding proteins; PLS, partial least square; RBP, K homology domain.

Based on the single-cell separation and Ti^4+^-IMAC capture strategy, we next performed quantitative nucleic acid–binding proteomics (supplemental Table S2) of mouse thymus and spleen from six mouse age stages to investigate the *in vivo* dynamics of NABPs activity during lifespan (1, 4, 12, 24, 48, and 72 weeks). The proportion of GO-annotated NABPs intensity ranged from 70% to 85% of all detected proteins, which was similar across all age stages for each organ (supplemental Table S4). The results of PCA and Pearson correlation analyses showed that these age stages were well separated, with biological replicates for each time point clustering more closely together than the replicates of other age stages (supplemental Fig. S4). Mice at different age stages exhibited a distinct and age-related nucleic acid–binding proteome profile. The number of DBPs and RBPs identified in each age stage varied, ranging from 1382 (1 week) to 1686 (12 weeks) in spleen and 1401 (1 week) to 1736 (12 weeks) in thymus (Fig. 4*A*).

To explore differences of NABP expression profiles across the lifespan, we generated a heatmap of the 1698 age-associated NABPs and searched for clusters of proteins showing parallel changes with age (Fig. 4*B*). Based on the partial least square analysis, we classified the age-associated NABPs into three age groups: young stage (1 week and 4 weeks), middle-aged stage (12 weeks and 24 weeks), and old mature stage (48 weeks and 72 weeks) (Fig. 4*C*). Volcano plots were generated to compare significantly changed proteins between young stage and old stage. Notably, the proteins most strongly associated with older age (Fig. 4*D*, left) were mesencephalic–astrocyte–derived neurotrophic factor (MANF), ribosomal protein family (Rpl23a, Rpl34, Rpl18), and telomerase-associated protein. MANF is expressed in immune cells and has an autocrine immunomodulatory function, promoting anti-inflammatory activation. Research showed that levels of MANF declined in aging flies, mice, and humans and that this reduction contributed to the age-related loss of tissue homeostasis in flies and mice (22). Ribosomal protein family plays an important role in the process of translation and leads to lifespan modulation. Its downregulation indicates the dysregulation protein synthesis with age, which was consistent with previous study (23). Telomerase is an enzyme responsible for the replication of the telomeric regions of chromosomal DNA. It is well known that the dysregulation expression activity of telomerase can serve as a signature for the replicative senescence of somatic cells and destabilization of their chromosomes. Consistent with previous research (24), our quantitative proteomics data also demonstrated that Pinx1(PIN2/TERF1-interacting telomerase inhibitor 1), Terf2 (telo-meric repeat-binding factor 1), and Terf2ip (telomeric repeat binding factor 2-interacting protein 1) were significantly increased, and Trir (telomerase RNA component-interacting RNase) were significantly decreased with aging (supplemental Table S5). These data verified the strong regulation between aging and NABPs and further proved the effectiveness of our established enrichment strategy.

### Immune Organ-Specific Pathway Profiling in the Aging Process

**Figure 5:**
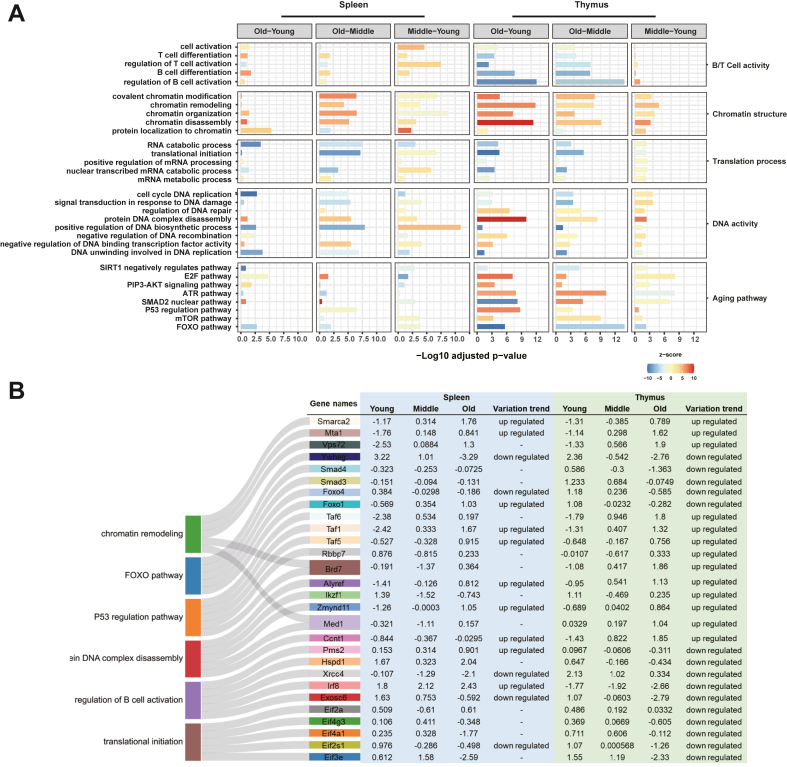
**Profiling immune organ-specific pathways during aging process.***A*, gene set variation analysis (GSVA) of NABPs at different age stages revealed the enriched pathways in spleen and thymus. *B*, significantly altered NABPs of different age stage involved in highly altered pathways (*p* < 0.05). NABP, nucleic acid–binding proteins.

To further investigate the NABPs aging signatures across mouse lifespan in immune organs, we performed a comprehensive functional analysis of the NABPome using GSVA method. We listed the significantly changed pathways and grouped them into five core functions to create a map of the age-related molecular network (Fig. 5*A*). During the aging process, spleen and thymus exhibited different functional changes over time. In the mouse thymus tissue, NABPs involved in functions such as ‘B/T cell activity’ and ‘translation process’ were dramatically downregulated, while those involved in the regulation of ‘chromatin structure’ and ‘DNA activity’ were strongly upregulated from middle stage to old stage. The typical aging pathways such as ‘FOXO pathway’, ‘SIRT1 pathway’, ‘ATR pathway’, and ‘mTOR pathway’ were all observed in the thymus. In the mouse spleen, the ‘B/T cell activity’, ‘chromatin structure’, ‘DNA activity’, and ‘aging pathway’ functions showed no obvious changes from middle stage to old stage. Previous studies have proved that the spleen controls the blood-borne immune response, which is an organ with the innate capacity to regenerate (25). Thus, we speculated that spleen plays an important role in maintaining immune defense functions and is less affected by aging. This phenomenon was further supported by the finding that the number of differentially expressed NABPs changes with age in spleen was dramatically less than that in the thymus (supplemental Fig. S5). In addition, many pathways such as ‘B/T cell activity’, ‘translation process’, ‘chromatin structure’, and ‘DNA activity’ were activated from young stage to middle stage in spleen rather than thymus, which may be related to the fact that thymus differentiates within the first week while the spleen’s maturing was slowly (26). These results suggest a unique aging response in spleen and thymus, as evidenced by distinct dynamic changes of NABPs.

Among the top six significantly altered pathways (fold change>2 or <0.5, *p* value<0.01), the NABPs that were continuously upregulated or downregulated with aging in thymus are shown in Fig. 5*B*. The normalized intensity represents the NBAPs binding activity across three different age stages of mice. In line with pathway altering, the proteins in spleen were not altered as dramatically as that in thymus. Taken together, using GSVA analysis, we deciphered the biological features of NABPome in mouse spleen and thymus over the lifespan, demonstrating that the aging process affects the two mouse organs, differently.

### WGCNA Analysis Helps Deciphering Interaction Networks for Aging

**Figure 6:**
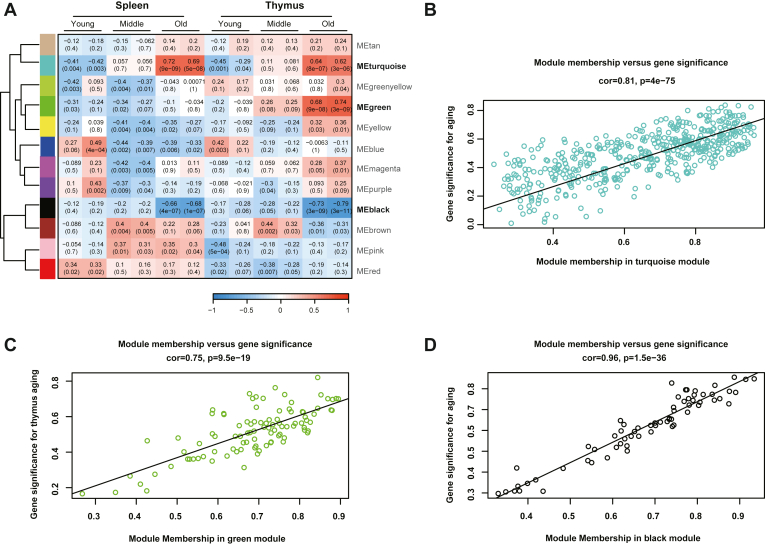
**The NABPs co-expression network**. *A*, module-trait associations of NABPs expression matrix. Each row corresponds to a module eigengene, column to a trait. Each cell contains the corresponding correlation and *p*-value. *B–D*, a scatterplot of gene significance (GS) for aging *versus* module membership (MM) in the (*B*) *turquoise*, (*C*) *green*, and (*D*) *black* module. NABP, nucleic acid–binding proteins.

The NABPs expression pattern was obtained after data preprocessing, composed of 1648 and 1524 NABPs from mouse thymus and spleen, respectively. Using R package WGCNA, we constructed a scale-free network (supplemental Fig. S5). A total of 12 modules were identified, with each color representing a different module (supplemental Table S6). A heat map was mapped about module–trait relationships according to the Spearman correlation coefficient to evaluate the association between each module and different age stage (Figs. 6*A* and S6). Among them, three modules, ‘turquoise’, ‘green’, and ‘black’ that had high correlation with the senile stage were selected as age-related modules. The turquoise modules including 482 NABPs were positively correlated with aging for spleen and thymus, respectively (Fig. 6*B*, correlation = 0.81, p = 4e-75). On the contrary, the black modules that included 65 proteins were negatively correlated with aging for both spleen and thymus (Fig. 6*C*, correlation =0.96, *p* = 1.5e-36). Interestingly, we found that the green module was positively correlated with aging specifically for thymus (Fig. 6*D*, correlation = 0.75, *p* = 9.5e-19), but not for spleen (correlation = 0.15, *p* = 0.23). Through WGCNA analysis, we discovered molecular networks associated with aging and found age-related modules.

### NABPs Driven Networks and Models for Aging

**Figure 7:**
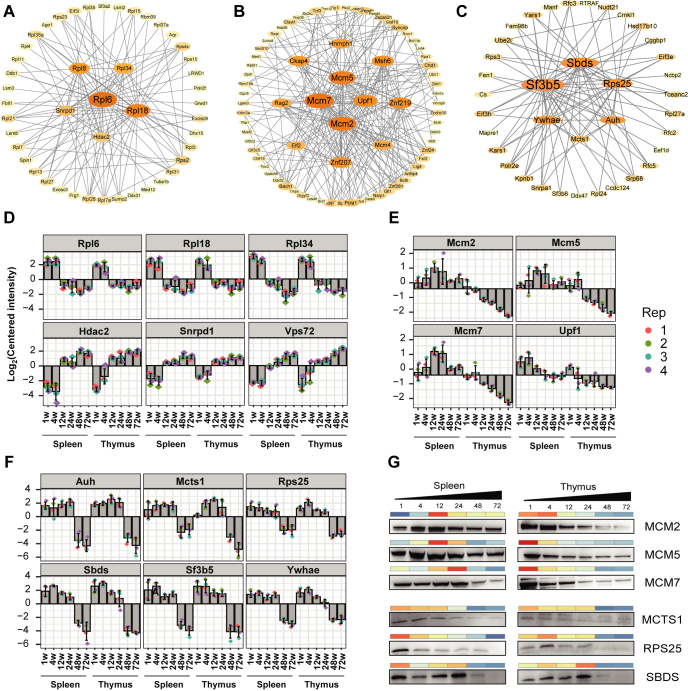
**The significant proteins that regulate aging in mouse spleen and thymus.***A–C,* differential expression proteins’ co-expression network in (*A*) *turquoise*, (*B*) *green*, and (*C*) *black* module. *D–F*, the hub protein expression of different mouse age stages in spleen and thymus in (*D*) *turquoise*, (*E*) *green*, and (*F*) *black* module. *G*, validation of hub proteins enriched by Ti^4+^-IMAC capture strategy. The bar above the blots corresponds to the quantification data of Western Blot (*red*/*blue* scale upregulated or downregulated, respectively). Ti^4+^-IMAC, titanium ion–immobilized metal-affinity chromatography.

We further constructed the interaction networks in ‘turquoise’, ‘green’, and ‘black’ modules and screened six, four, and six proteins with highest connectivity as hub proteins, respectively (Fig. 7, *A–C*). These hub NABPs that had a strong degree of association in the interaction network play more important roles in regulating aging than other NABPs. Subsequently, we combined the protein interaction networks with the quantitative proteome dataset to obtain the change tendency of hub NABPs during aging (Fig. 7, *D–F*). To validate these hub proteins, we performed Western blotting analysis (Figs. 7*G* and S7). Our immunoassay results of these proteins at NABPs level were basically consistent with LC-MS/MS-based proteome analysis. In particular, MCM2 (minichromosome maintenance complex 2), MCM5, MCM7, multiple copies in T-cell lymphoma-1, RPS25 ribosomal protein S25, and Shwachman-Bodian-Diamond Syndrome were successfully confirmed (Figs. 7*G* and S7).

Among the 482 NABPs in the turquoise module, 42 proteins were selected to construct PPI network, and top six proteins with the highest connectivity related to spleen and thymus aging were screened as hub nodes. Among them, RPL6 (ribosomal protein L6), RPL18, and RPL34 were downregulated, while HDAC2, small nuclear ribonucleoprotein D1 polypeptide, and vacuolar protein sorting 72 homolog were upregulated with aging (Fig. 7*D*). Ribosomal protein family play an important role in regulating translational activity, especially in controlling the rate of protein synthesis. The decrease of protein homeostasis, including the slowdown of protein synthesis and degradation, is a major hallmark of aging. A recent study in the worm *Caenorhabditis elegans* confirmed that the degradation rates of studied proteins decreased 40% with aging. Interestingly, the most prominent decrease was found for ribosomal proteins and proteins participating in translation regulation (27). The downregulation of ribosomal protein in our result illustrates the slowdown of protein synthesis, which proved the point that there is a causative relationship between the regulation of protein homeostasis and aging.

Among the 97 NABPs in the green module, 61 proteins were selected to construct PPI network, and top four proteins related to thymus aging were specifically screened as hub proteins (Fig. 7*B*). Interestingly, the DNA binding ability of MCM2-7 proteins decreased with aging in thymus, while first rise then fall with aging in spleen (Fig. 7*E*). MCM proteins form a hexamer, which functions as replicative helicase, and are proliferation markers in cell lines. Recent studies showed that (28) less frequent divisions of fibroblasts with increasing age correlate with low MCM protein levels. Correspondingly, we found that MCM complex was downregulated in thymus and spleen with aging, which indicated that the inevitably decreased cell division rates with aging.

Among the 65 NABPs in the black module, 61 proteins were selected to construct PPI network, and top six proteins related to thymus aging were specifically screened as hub proteins (Fig. 7, *C* and *F*), which were all downregulated. Although these proteins have not been reported to be associated with aging, their expression is closely related to metabolism and oxidative stress. monocarboxylate transporter 1 is partially responsible for lactate uptake by the germ cells (29). Methylglutaconyl-CoA hydratase participates in catabolism of branched amino acids and catalyzes the fifth step in the leucine degradation pathway (30). Splicing factor 3B subunit 5 might involve in pyrimidine metabolism pathways (31). In addition, knockdown of Shwachman-Bodian-Diamond Syndrome in HeLa cells and TF-1 myeloid cells resulted in a significant increase in reactive oxygen species, subsequently reduced cell growth (32). The downregulation of these proteins, regardless of in thymus and spleen, reveals that there are common NABPs changes during aging process in each immune organ. And these changes are due to inevitable extracellular factors like oxidative damage and calorie intake.

## Discussion

In the last decade, lots of methodologies have been designed to study the nucleic acid–binding proteome composition of cells using different strategies. Several of these strategies are complex to operate, including the genomic design (*e.g.,* catTFRE) (33), the DNA or RNA mediation (*e.g.,* Dm-ChP or RICK) (34), or the capture of specific genomic repetitive regions (*e.g.,* ASO) (35). Several purifying methods bring a remarkable abundance of contaminants, such as antibodies, that can mask the detection of low-abundant NABPs (*e.g.,* CHIP or RIP) (36). Although there are some flexible approaches satisfied efficiently enrich and quantify the complete suite of RBPs (*e.g.,* XRNAX, OOPS), these methods are mainly developed for cell lines, not for tissues or organs.

The NABPs enrichment pipeline for mouse organs presented in this study is a powerful method for NABPome profiling. The experimental protocol and data highlighted its simplicity, robustness, and utility in different type of organs. In our method, living cells were obtained from fresh solid organs by mechanical dissociation and collagenase digestion. TRIZOL denaturation was performed after formaldehyde crosslinking, and nucleic acid-protein complex was extracted by Ti^4+^-IMAC. Approximately, 2000 proteins were quantified in each organ (kidney, lung, spleen, and thymus), the proportion of GO-annotated NABPs reached 70 to 90%. We believe that Ti^4+^-IMAC–based NABPs enrichment methodology is suitable for various solid organs and has many advantages and unique merits. First, the experimental protocol is simple and straightforward. Second, the method is selective and reliable, allowing the measurement of low levels of NABPs. Most importantly, weak nucleic acid–protein interactions can be maintained through *in situ* crosslinking by formaldehyde. However, it should be noted that the sample pretreatment will lead to the RNA degradation, which resulting in the reduction of NABPs yield. Therefore, the entire process should be performed on ice. By optimizing the concentration and crosslinking time of formaldehyde, the RNA recovery rate can reach about 80%.

Recent studies found that altered chromatin structure, accumulated DNA damage, and changes in global transcriptional programmers are all hallmarks of aging (37). We therefore hypothesized that the proteins-binding capability to DNA/RNA in this process is highly related to aging and age-related degenerative diseases. In order to better understand and characterize age-related NABPome, we captured the NABPs of natural aging mouse model by using single cell preparation and Ti^4+^-IMAC technology-based proteomics approach. In this work, through comparative analysis of global NABPome in mouse spleen and thymus at 1, 4, 12, 24, 48, and 72 weeks, we observed a number of organ-specific biological processes (Fig. 5). Not only several aging-associated pathways (*e.g*., SIRT1 pathway, P53 pathway, FOXO pathway) consistent with previous studies (38) were observed, we also revealed novel aging-related changes in chromatin composition and translational process (*e.g.,* chromatin remodeling, chromatin disassembly, and translational initiation) that were underestimated by conventional proteomic analysis (39). Interestingly, we identified distinct aging features between thymus and spleen. The spleen NABPome was rather constant during the mouse lifespan and displayed a slight correlation with aging pathway, which may reflect the older spleen maintaining a young phenotype and continuously exhibiting the role of maintaining important immune and defense activities. In contrast, thymus NABPome exhibited temporal dynamics of expression during the mouse lifespan, which is speculated to be closely related to thymus degenerate process. It is also worth to notice that the key metabolism pathways (*e.g.,* amino acid metabolic, ATP metabolic) were suppressed with aging, while oxidative stress (*e.g.,* external stimulus, ATP metabolic) was activated in both spleen and thymus, indicating inevitable connections among environment and aging independent of organs.

WGCNA is a systems biology method for describing the pairwise relationships among interacting proteins of multiple samples. It can cluster proteins and form modules by similar protein expression patterns and analyze the relationship between modules and specific features (*e.g.,* aging of specific organs). To gain more insight into the relationship between the aging and the dysregulated NABPs, we performed the WGCNA analysis of our proteomics data. We selected the proteins from modules that are highly correlated with age stage and depicted NABPs-driven networks and models for aging. Specifically, the ‘turquoise’, and ‘black’ modules were positively and negatively associated with aging, respectively, for both thymus and spleen. However, the ‘green’ modules were negatively associated with thymus only. We assumed that the age-related binding affinity changes of NABPs can be attributed to genomic instability with aging according to recent research (40). Following the further Western blot verification, 10 hub NABPs were verified, which were considered as candidates for investigating aging and associated mechanisms.

Currently, most of the studies on aging and NABPs are correlational (41), and only limited proteomic research attempted to discover the NABP-mediated regulatory influences in cellular aging and related disease processes. A previous study has linked chromatin proteomes to aging-related features of each organ *via* nuclear protein–extraction protocol (8). However, they missed the opportunity to explain which protein or what protein functions that play important role in aging. With our systematic proteomic analysis of NABPs of natural aging mouse model, Ti^4+^-IMAC capture method identified the intimate and dynamic nucleic acid–protein interactions and key regulators of aging. It could enrich low-abundant NABPs and remove high abundance protein interferences. In particular, the developed strategy is suitable for various organ tissues, which demonstrated high NABPs extraction yield and ideal reproducibility. However, our study did not provide detailed information on the mechanism of the NABPs-mediated aging process. Taken together, our findings suggested that NABPs recruited by target genes was regulated by aging process. Our proteomic analysis strategy provides a pipeline for further exploring NABPs signatures that play important functions in other physiological circumstance. Although further functional verification is needed for the mechanism of aging, these hub NABPs are promising targets for aging and related diseases.

## Conclusion

In this study, we developed a simple, robust, and selective strategy for the comprehensive analysis of NABPome, which captured the intimate and dynamic *in situ* nucleic acid–protein interactions. Single cell preparation and Ti^4+^-IMAC-based proteomic technology was successfully applied to the discovery of aging-related NABPs. Unique aging features in mouse spleen and thymus were discovered, and a co-expression NABPs network was constructed. Hub proteins that acted as essential components in the process of aging through binding to their target genes or RNA were revealed and validated. The developed strategy expanded the knowledge of aging and established the correlation between NABPs and aging, which will provide a foundation for the understanding of how NABPs execute their regulatory functions during aging.

## Data Availability

The mass spectrometry proteomics data and the search data by MaxQuant (version 1.6.1.0) have been deposited to the ProteomeXchange Consortium *via* the PRIDE (42) partner repository with the dataset identifier PXD026035.

## Conflict of interest

The authors declare no competing interests.
