# Supplementary material for: Deciphering Nucleic Acid Binding Proteome of Mouse Immune Organs Reveals Hub Proteins for Aging
Source: Mol Cell Proteomics. 2023 Jun 28;22(8):100611. doi: 10.1016/j.mcpro.2023.100611 (PMC10412848; doi:10.1016/j.mcpro.2023.100611)
Supplement: Supplemental Information [file mmc1.docx]

Supporting information for

**Deciphering nucleic acid binding proteome of mouse immune organs reveals hub proteins for aging**

Huiyu Wang ^a, †^, Yan Zhang ^b, †^, Zeyuan Wang ^a^, Lu Zhang ^a^, Miao Guo ^a^,

Chengxi Cao ^c^, Hua Xiao ^a,^*

^a^ State Key Laboratory of Microbial Metabolism, Joint International Research Laboratory of Metabolic & Developmental Sciences, School of Life Sciences and Biotechnology, Shanghai Jiao Tong University, Shanghai, 200240, China.

^b^ School of Pharmacy, Shanghai Jiao Tong University, Shanghai, 200240, China

^c^ Department of Instrument Science and Engineering, School of Electronic Information and Electrical Engineering, Shanghai Jiao Tong University, Shanghai 200240, China

*^*^* Corresponding Author:

Prof. Dr. Hua Xiao, Email address: [huaxiao@sjtu.edu.cn](mailto:huaxiao@sjtu.edu.cn)

^†^These authors contributed equally as first authors to this work.

**This supporting information contains:**

**Table S1.** Detected classical DBPs and RBPs in mouse organs

**Table S2.** GO annotated NABPs identification in cell lysate and Ti4+-IMAC Enriched group

**Table S3.** Differentially expressed proteins among different organs

**Table S4.** NABPs identification in mouse spleen and thymus at 6 aging stages

**Table S5.** Telomerase related NABPs identified in our experiments

**Table S6.** Number distribution of NABPs in different modules after WGCNA analysis

**Table S7.** Coefficient of variation of the normalized LFQ intensity obtained from different tissues

**Table S8.** Coefficient of variation of the normalized LFQ intensity obtained from different aging stage

**Figure S1.** Dynamic range plot of proteins identified in Ti4+-IMAC capture group and total tissue lysate group of different mouse organs.

**Figure S2**. NABPs enrichment degree of different mouse organs.

**Figure S3.** Comparative and quantitative proteomics of total proteins and NABPs in mouse organs.

**Figure S4.** Reproducibility and consistency analysis of NABP enrichment.

**Figure S5.** Scale-free network construction using WGCNA.

**Figure S6**. Co-expression blocks of NABPs.

**Figure S7**. Biological replicates for validation of hub protein responses to aging enriched by Ti^4+^-IMAC capture strategy.

**Table S1. Classical DBPs and RBPs in mouse organs**

| Nucleosome remodelling and deacetylase family  (DBPs) | K homology domain family  (RBPs) |
| --- | --- |
| Mbd3 | Pnpt1 |
| Hdac1 | Hdlbp |
| Chd4 | Hnrnpk |
| Gata3 | Pcbp1 |
| Mta1 | Fxr1 |
| Mta3 | Sf1 |
| Rbbp4 | Krr1 |
| Hdac2 | Tdrkh |

**Table S2. GO annotated NABPs identification in cell lysate and Ti^4+^-IMAC Enriched group**

| Sample | Total protein | NABP | NABP Intensity  Percent | NABP Number  Percent |
| --- | --- | --- | --- | --- |
| Thymus Cell lysate | 3458 | 1091 | 51% | 31% |
| Thymus Ti^4+^-IMAC Enriched group | 2181 | 1279 | 86% | 60% |
| Spleen Cell lysate | 3638 | 1048 | 45% | 29% |
| Spleen Ti^4+^-IMAC Enriched group | 2543 | 1335 | 77% | 54% |
| Kidney Cell lysate | 2626 | 427 | 27% | 16% |
| Kidney Ti^4+^-IMAC Enriched group | 2169 | 1317 | 64% | 60% |
| Lung Cell lysate | 2761 | 601 | 22% | 22% |
| Lung Ti^4+^-IMAC Enriched group | 1749 | 835 | 80% | 50% |

**Table S3. Differentially expressed proteins among different organs**

|  | Kidney/ Lung | Kidney/ Spleen | Kidney/  Thymus | Lung/ Spleen | Lung/  Thymus | Spleen/ thymus |
| --- | --- | --- | --- | --- | --- | --- |
| Downregulated | 732 | 251 | 370 | 1790 | 1562 | 398 |
| No significance | 1171 | 3218 | 2625 | 1213 | 1248 | 2510 |
| Upregulated | 1727 | 161 | 635 | 627 | 820 | 722 |

**Table S4. NABPs identification in mouse spleen and thymus at 6 stages**

| Groups | NABPs number | NABPs Intensity  Percent (%) | NABPs Number  percent (%) |
| --- | --- | --- | --- |
| spleen-1w | 1382 | 81.4603 | 51.1155 |
| spleen-4w | 1444 | 83.0727 | 53.1192 |
| spleen-12w | 1653 | 75.3298 | 50.4255 |
| spleen-24w | 1613 | 78.6672 | 54.1214 |
| spleen-48w | 1686 | 77.8974 | 52.3813 |
| spleen-72w | 1653 | 72.4837 | 56.4353 |
| thymus-1w | 1401 | 85.3178 | 55.4926 |
| thymus-4w | 1638 | 79.8755 | 49.5932 |
| thymus-12w | 1736 | 76.9947 | 45.9341 |
| thymus-24w | 1693 | 76.5787 | 48.2704 |
| thymus-48w | 1687 | 79.1295 | 49.6621 |
| thymus-72w | 1598 | 80.1312 | 52.2240 |

Note: The NABPs Intensity Percent was calculated as follows: (The sum of GO annotated NABPs Intensity / The sum of Ti^4+^-IMAC captured proteins Intensity) ×100%. The NABPs Number Percent was calculated as follows: (The number of GO annotated NABPs / The number of Ti^4+^-IMAC captured proteins) ×100%.

**Table S5. Telomerase related NABPs identified in our experiments**

| \| Protein names \| Gene name \| Fold change spleen \| Fold change thymus \| P-value spleen \| P-value thymus \| \| --- \| --- \| --- \| --- \| --- \| --- \| \| PIN2/TERF1-interacting telomerase inhibitor 1 \| Pinx1 \| 2.7544 \| 9.8102 \| 0.0000 \| 0.0081 \| \| Telomerase-binding protein EST1A \| Smg6 \| -1.6360 \| -1.4853 \| 0.4328 \| 0.5351 \| \| Telomerase RNA component interacting RNase \| Trir \| -2.2620 \| -2.1761 \| 0.0241 \| 0.0939 \| \| Telomerase Cajal body protein 1 \| Wrap53 \| 2.1083 \| 1.1139 \| 0.5886 \| 0.9929 \| \| H/ACA ribonucleoprotein complex subunit DKC1 \| Dkc1 \| 2.0477 \| 1.5122 \| 0.1089 \| 0.2583 \| \| H/ACA ribonucleoprotein complex subunit 2 \| Nhp2 \| -3.1867 \| -42.0880 \| 0.0000 \| 0.0000 \| \| Telomeric repeat-binding factor 1 \| Terf1 \| 2.8684 \| 2.6580 \| 0.0352 \| 0.0004 \| \| Telomeric repeat-binding factor 2 \| Terf2 \| 2.2406 \| 2.4626 \| 0.0183 \| 0.0295 \| \| Telomeric repeat-binding factor 2-interacting protein 1 \| Terf2ip \| 1.4665 \| 1.8148 \| 0.0118 \| 0.1567 \| \| TERF1-interacting nuclear factor 2 \| Tinf2 \| 1.1148 \| 0.8988 \| 0.3684 \| 0.6648 \| |
| --- | --- | --- | --- | --- | --- | --- | --- | --- | --- | --- | --- | --- | --- | --- | --- | --- | --- | --- | --- | --- | --- | --- | --- | --- | --- | --- | --- | --- | --- | --- | --- | --- | --- | --- | --- | --- | --- | --- | --- | --- | --- | --- | --- | --- | --- | --- | --- | --- | --- | --- | --- | --- | --- | --- | --- | --- | --- | --- | --- | --- | --- | --- | --- | --- | --- | --- |

**Table S6.** **Number distribution of NABPs in different modules after WGCNA analysis**

| Modules | Black | Blue | Brown | Green | Greenyellow | Megta |
| --- | --- | --- | --- | --- | --- | --- |
| NABPs number | 65 | 344 | 133 | 97 | 24 | 25 |
| Module | Pink | Purple | Red | Tan | Turquoise | Yellow |
| NABPs number | 60 | 24 | 66 | 20 | 482 | 110 |

**Table S7. Coefficient of variation of the normalized LFQ intensity obtained from different tissues**

| **Kidney** | | |
| --- | --- | --- |
| Ti^4+^-IMAC enriched_RP1 | Ti^4+^-IMAC enriched_RP2 | Ti^4+^-IMAC enriched_RP3 |
| 10.1 | 11.893 | 11.795 |
| Cell lysate_RP1 | Cell lysate_RP2 | Cell lysate_RP3 |
| 10.712 | 10.648 | 10.579 |
| **Lung** | | |
| Ti^4+^-IMAC enriched_RP1 | Ti^4+^-IMAC enriched_RP2 | Ti^4+^-IMAC enriched_RP3 |
| 11.599 | 11.439 | 11.377 |
| Cell lysate_RP1 | Cell lysate_RP2 | Cell lysate_RP3 |
| 11.152 | 11.123 | 11.049 |
| **Spleen** | | |
| Ti^4+^-IMAC enriched_RP1 | Ti^4+^-IMAC enriched_RP2 | Ti^4+^-IMAC enriched_RP3 |
| 11.616 | 11.64 | 11.438 |
| Cell lysate_RP1 | Cell lysate_RP2 | Cell lysate_RP3 |
| 10.87 | 10.823 | 10.919 |
| **Thymus** | | |
| Ti^4+^-IMAC enriched_RP1 | Ti^4+^-IMAC enriched_RP2 | Ti^4+^-IMAC enriched_RP3 |
| 11.888 | 11.776 | 11.8 |
| Cell lysate_RP1 | Cell lysate_RP2 | Cell lysate_RP3 |
| 11.108 | 11.044 | 11.036 |

**Table S8. Coefficient of variation of the normalized LFQ intensity obtained from different aging stage**

| Spleen | | | | Thymus | | | |
| --- | --- | --- | --- | --- | --- | --- | --- |
| 1W_RP1 | 1W_RP2 | 1W_RP3 | 1W_RP4 | 1W_RP1 | 1W_RP2 | 1W_RP3 | 1W_RP4 |
| 12.543 | 11.030 | 12.688 | 12.914 | 11.593 | 11.516 | 11.588 | 11.670 |
| 4W_RP1 | 4W_RP2 | 4W_RP3 | 4W_RP4 | 4W_RP1 | 4W_RP2 | 4W_RP3 | 4W_RP4 |
| 11.424 | 11.496 | 11.515 | 11.218 | 10.318 | 11.591 | 10.705 | 11.852 |
| 12W_RP1 | 12W_RP2 | 12W_RP3 | 12W_RP4 | 12W_RP1 | 12W_RP2 | 12W_RP3 | 12W_RP4 |
| 10.333 | 10.402 | 10.483 | 10.581 | 10.814 | 10.774 | 10.684 | 10.722 |
| 24W_RP1 | 24W_RP2 | 24W_RP3 | 24W_RP4 | 24W_RP1 | 24W_RP2 | 24W_RP3 | 24W_RP4 |
| 10.282 | 10.518 | 10.967 | 10.725 | 11.259 | 10.940 | 10.694 | 11.252 |
| 48W_RP1 | 48W_RP2 | 48W_RP3 | 48W_RP4 | 48W_RP1 | 48W_RP2 | 48W_RP3 | 48W_RP4 |
| 11.132 | 11.279 | 11.355 | 11.244 | 11.367 | 11.436 | 11.845 | 11.945 |
| 72W_RP1 | 72W_RP2 | 72W_RP3 | 72W_RP4 | 72W_RP1 | 72W_RP2 | 72W_RP3 | 72W_RP4 |
| 10.209 | 11.356 | 11.365 | 11.552 | 10.519 | 11.857 | 10.177 | 10.036 |


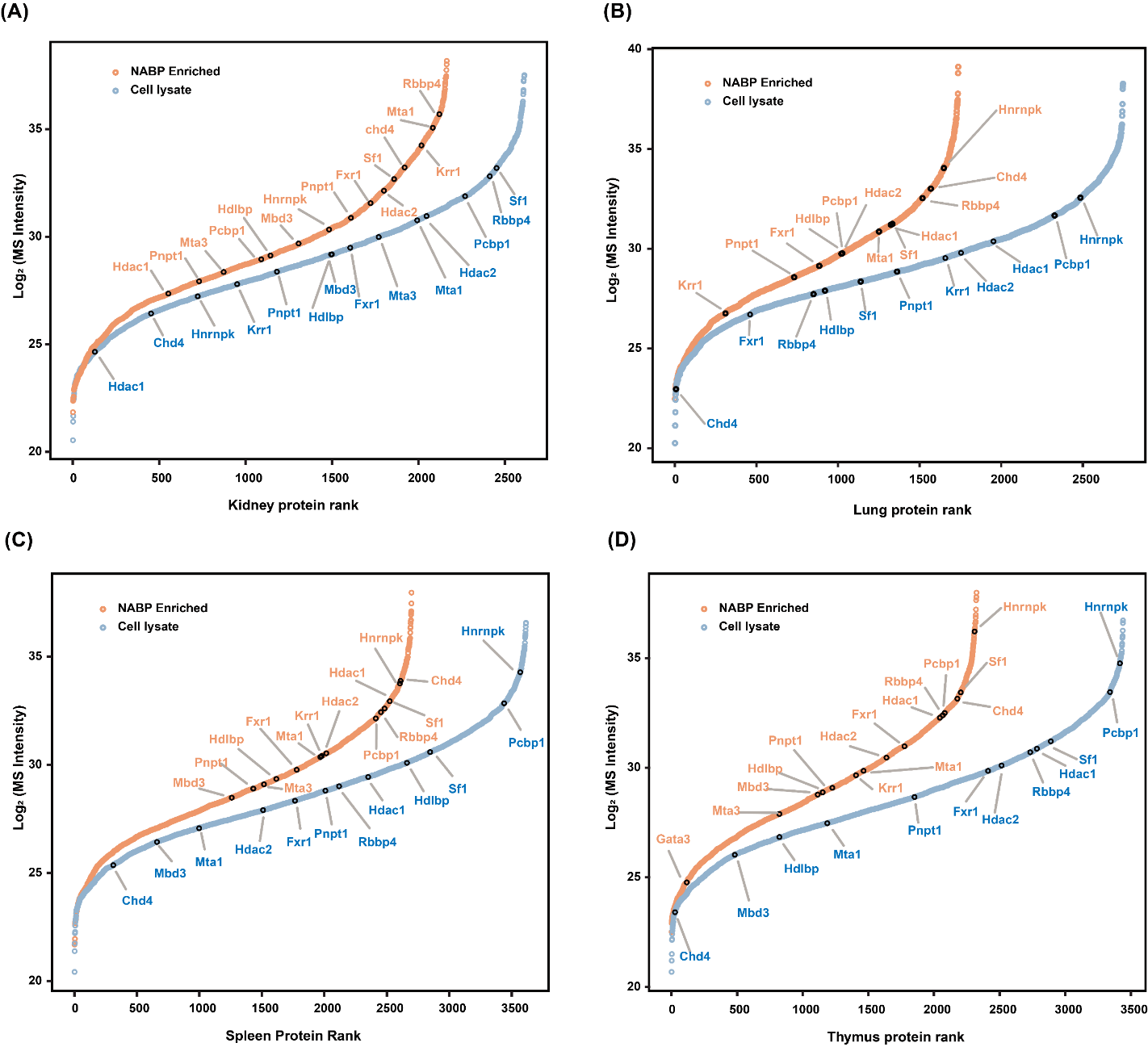


**Figure S1.** Dynamic range plot of proteins identified in Ti^4+^-IMAC capture group and total tissue lysate group of (A) Kidney, (B) Lung, (C) spleen, and (D) thymus. Nucleosome remodeling and deacetylase and K-homology type protein family are listed and sorted by their abundance (Log_2_ MS intensity).


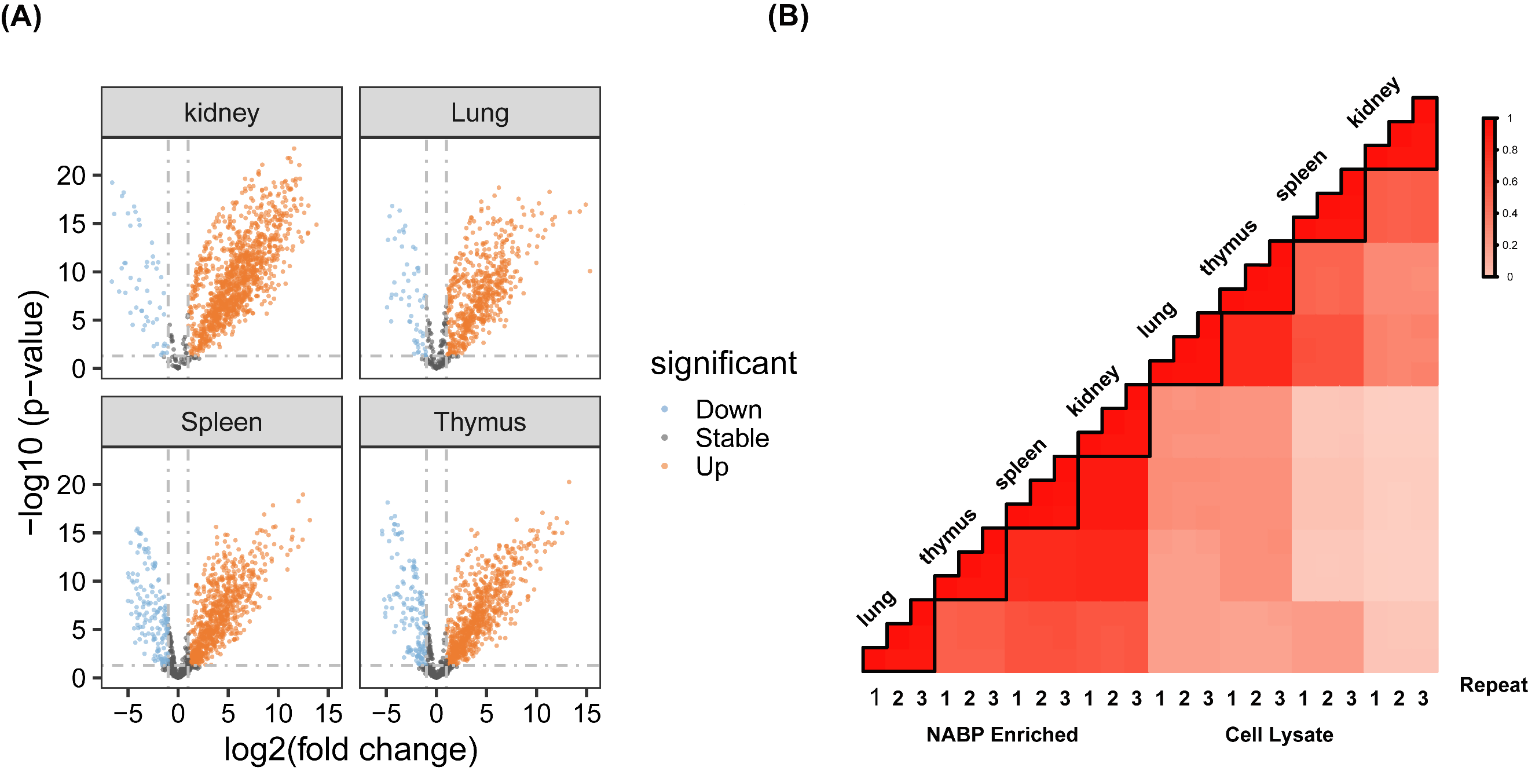


**Figure S2**. NABPs enrichment degree of different mouse organs. (A) Volcano plots of NABPs enriched by Ti^4+^-IMAC capture method compared with tissue lysate in kidney, lung, spleen, and thymus. (B) Pearson correlation coefficient showing the relationship between the Ti4+-IMAC capture group and total tissue lysate group..


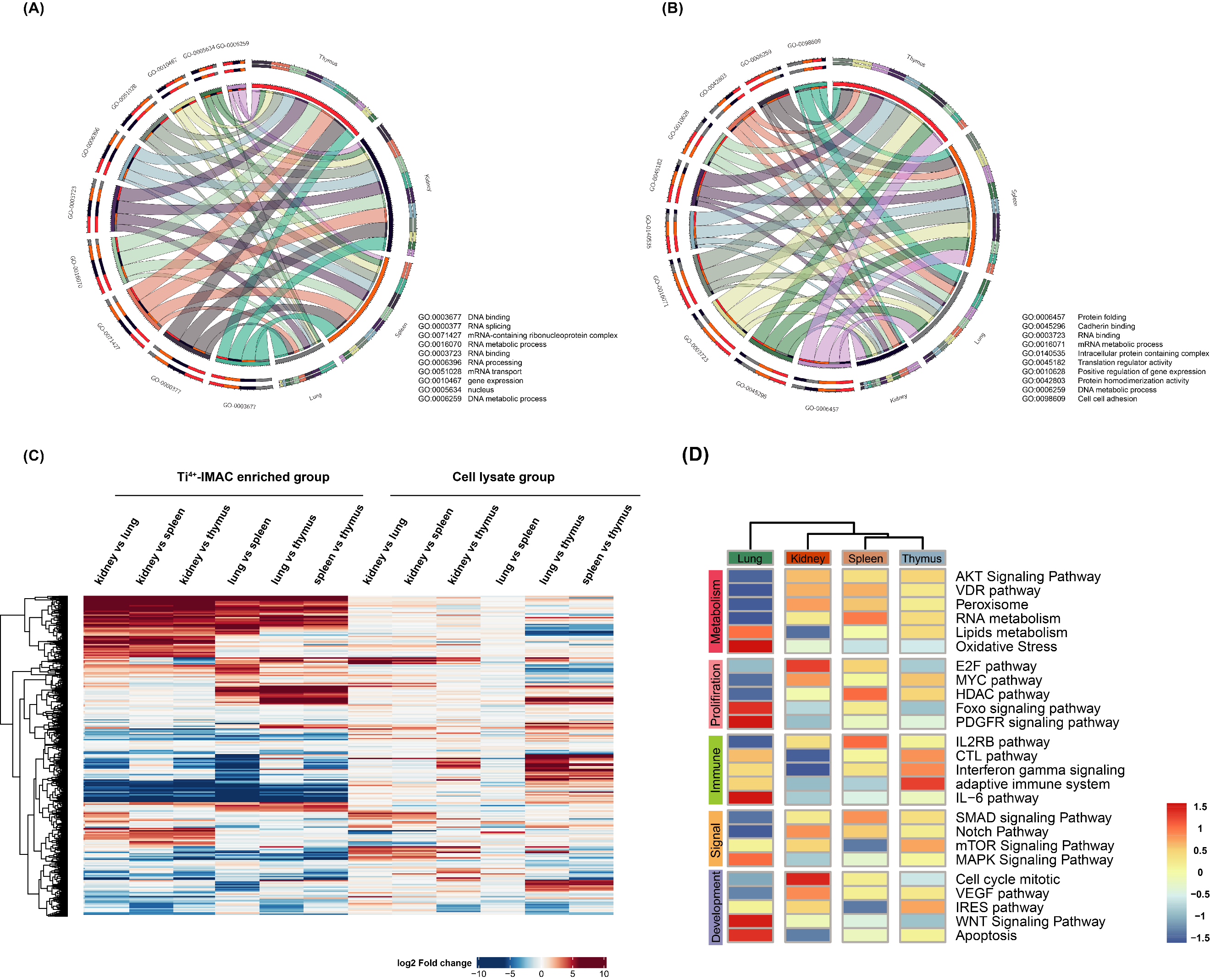


**Figure S3.** Comparative and quantitative proteomics of total proteins and NABPs in mouse organs. (A) GO function integration of proteins in Ti^4+^-IMAC capture group (top 10 abundant GO functions). (B) GO function integration in total tissue lysate group (top 10 abundant GO functions). (C) Heatmap of protein abundance foldchange in Ti4+-IMAC enrich group and total protein of different tissues. (D) Heat map of significantly altered pathways in different mouse organs. Color of each cell represents the average ssGSEA enrichment scores of that subtype; red denotes activation and blue denotes inhibition.


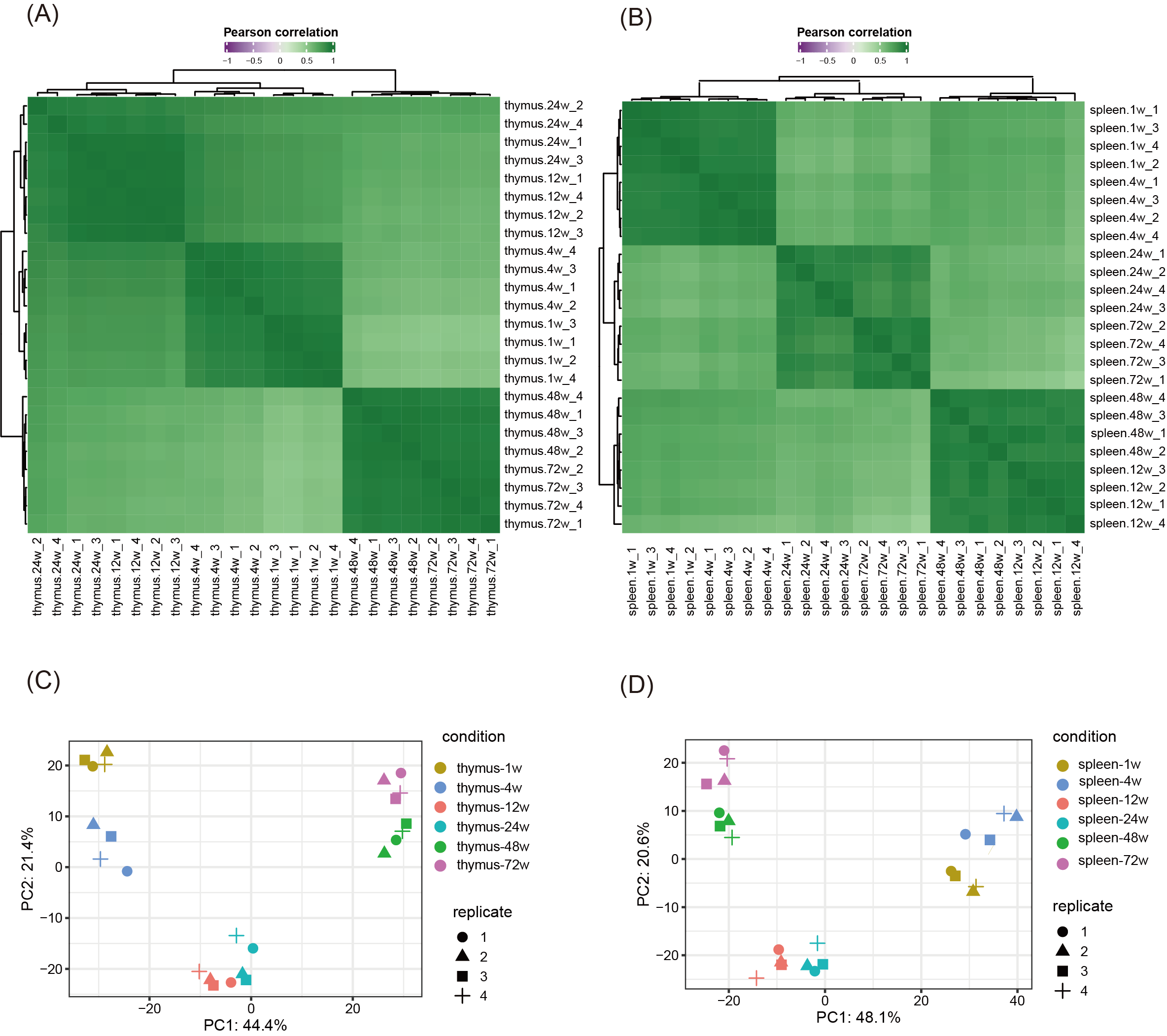


**Figure S4**. Reproducibility and consistency analysis of NABP enrichment method. Pearson correlation coefficient showing the relationship between the different age stage of enriched nucleic acid binding proteome in (A) thymus and (B) spleen. Principal component analysis (PCA) of (C) thymus and (D) spleen nucleic acid binding proteomics datasets in different age stage. Each data point represents a single biological replicate (n = 4). Color subgroups represent each mouse lifespan stage at 1, 4, 12, 24, 48 and 72 weeks, respectively.


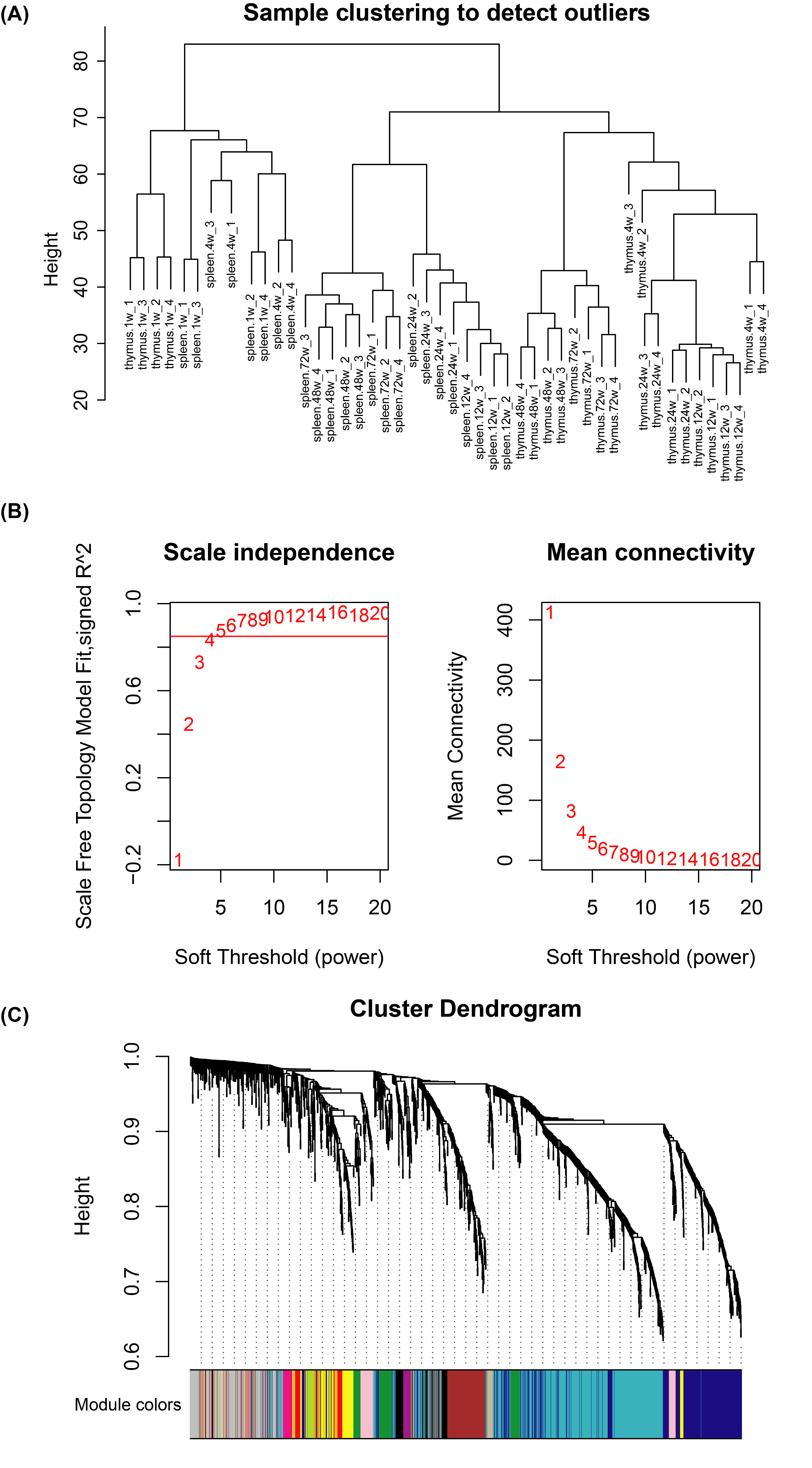


**Figure S5**. Scale-free network construction using WGCNA. (A) Clustering dendrogram of different age stage sample in thymus and spleen based on their Euclidean distance. (B) Analysis of the scale-free index and mean connectivity for various soft-threshold powers. (C) Dendrogram of all differentially expressed genes clustered based on the measurement of dissimilarity


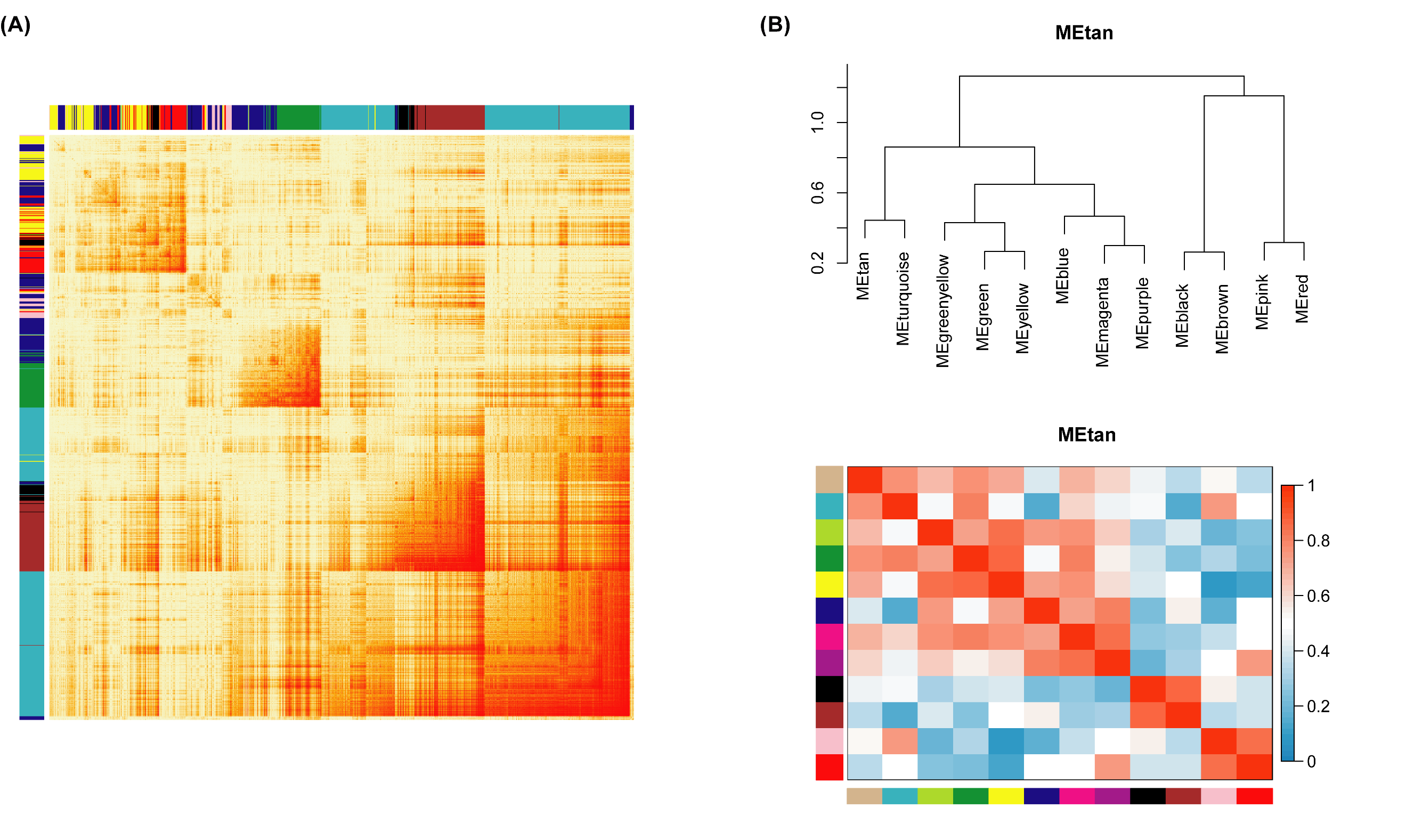
**Figure S6**. Co-expression blocks of NABPs. (A)Twelve co-expression modules of NABPs were identified in 6 different stages of thymus and spleen. Pearson's correlation coefficient of 0.4 was used as a cutoff, and Kendall's Tau test was applied to test for significance. (B) Eigengene dendrogram and eigengene adjacency plot.


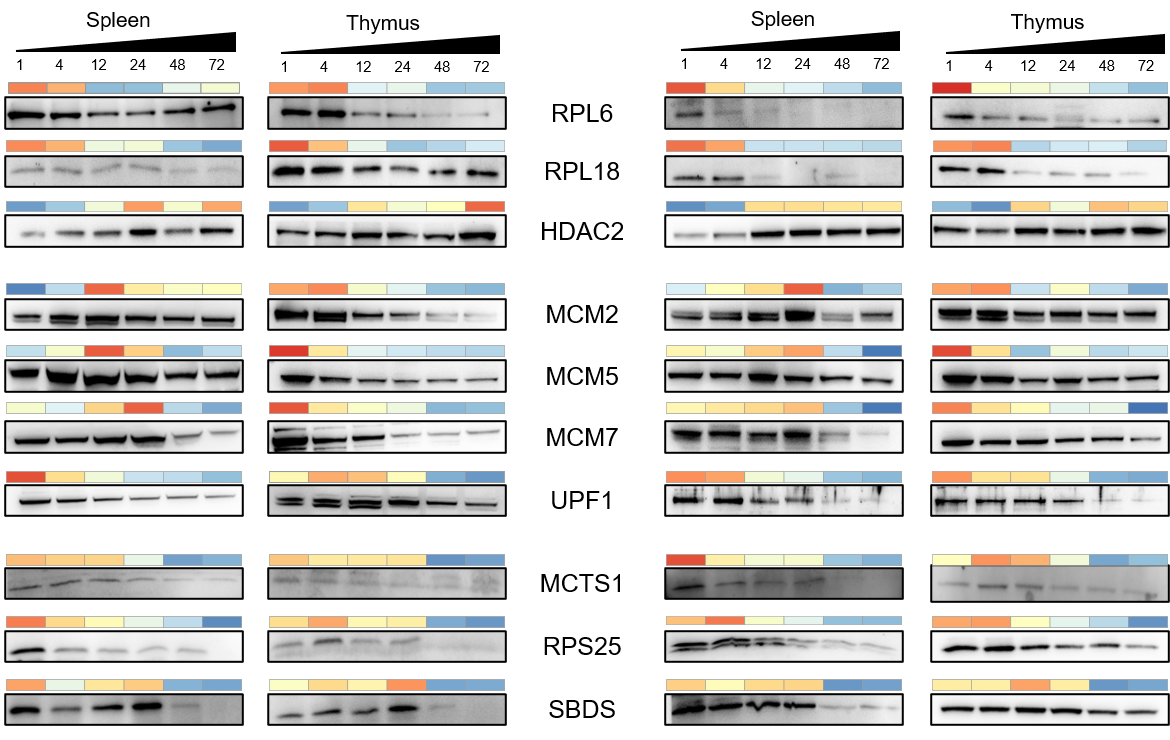


**Figure S7**. Biological replicates for validation of hub protein responses to aging enriched by Ti^4+^-IMAC capture strategy. The bar above the blots corresponds to the quantification data of Western Blot (red/blue scale up- or downregulated, respectively)
